# Clinical characteristics and antibiotic treatment of peritoneal dialysis-associated peritonitis caused by *Pseudomonas* species: a review of 15 years’ experience from southern China

**DOI:** 10.1128/spectrum.00096-24

**Published:** 2024-05-02

**Authors:** Xiao Dong, Chao Xie, Chunyan Yi, Peiyi Ye, Hongjian Ye, Qunying Guo, Fengxian Huang, Yao-Zhong Kong, Xiao Yang

**Affiliations:** 1 Department of Nephrology, The First Affiliated Hospital, Sun Yat-sen University, Key Laboratory of Nephrology, National Health Commission and Guangdong Province, Guangdong, Guangdong, China; 2 Department of Nephrology, The First People's Hospital of Foshan, Foshan, Guangdong, China; Central Texas Veterans Health Care System, Temple, Texas, USA

**Keywords:** peritoneal dialysis, *Pseudomonas* peritonitis, antibiotics, drug resistance

## Abstract

*Pseudomonas* can lead to peritoneal dialysis-associated peritonitis, which is characterized by a poor prognosis, such as a substantial failure rate and a high death rate. This study aimed to provide an overview of *Pseudomonas* peritonitis’s clinical features, the regimens of antibiotic, antibiotic resistance, and outcomes in peritoneal dialysis (PD) patients. This study observed patients with *Pseudomonas* peritonitis in two large PD centers in South China from January 2008 to December 2022. The demographics, symptomatology, antibiotics regimens, resistance to common antibiotics, and clinical outcomes of all included patients were reviewed. A total of 3,459 PD patients were included, among them 57 cases of peritonitis caused by *Pseudomonas*, including 48 cases (84.2%) of *Pseudomonas aeruginosa*. The incidence rate of *Pseudomonas* peritonitis was 0.0041 episode per patient-year. Of them, 28.1% (16 cases) of the patients were accompanied by exit site infection (ESI), and all had abdominal pain and turbid ascites at the time of onset. The most commonly used antibiotic combination was ceftazidime combined with amikacin. Approximately 89% of *Pseudomonas* species were sensitive to ceftazidime, and 88% were sensitive to amikacin. The overall primary response rate was 28.1% (16 patients), and the complete cure rate was 40.4% (23 patients). There was no significant difference in the complete cure rate of peritonitis using three and other antibiotic treatment regimens (44.8% vs 46.4%; *P* = 0.9). The successful treatment group had higher baseline albumin level (35.9 ± 6.2; *P* = 0.008) and residual urine volume (650.7 ± 375.5; *P* = 0.04). Although the incidence of peritonitis caused by *Pseudomonas* was low, the symptoms were serious, and prognosis was very poor. *Pseudomonas* was still highly susceptible to first-line antibiotics currently in use against Gram-negative bacteria. Patients with successful treatment had higher albumin levels and higher urine output.

**IMPORTANCE:**

Although the incidence of peritoneal dialysis-associated peritonitis caused by *Pseudomonas* is very low, it seriously affects the technique survival of peritoneal dialysis patients. However, there are few studies and reports on *Pseudomonas* peritonitis in the Chinese mainland area. Therefore, the purpose of this study is to describe the clinical characteristics, the regimens of antibiotic, drug resistance, and outcome of peritoneal dialysis patients in southern China in the past 15 years and summarize the clinical experience in the treatment of *Pseudomonas* peritonitis.

## INTRODUCTION

Despite a global decline in the incidence of peritonitis, it remains the major cause of peritoneal dialysis (PD) failure (1). According to our earlier research, up to 33% of death-censored technique failures in the first year were caused by peritonitis (2). *Pseudomonas* is a common non-fermentative aerobic Gram-negative bacteria in the clinic, which is widely distributed in the living environment and can cause a variety of infections (3). Although the incidence of peritoneal dialysis-associated peritonitis (PDAP) caused by *Pseudomonas* was low, treatment was often ineffective, and cure rates tend to be only 20% in a UK study, significantly impacting the technique survival of PD patients (4). Among them, *Pseudomonas aeruginosa* easily forms a biofilm in the catheter, which increases antibiotic resistance and probability of recurrence (5). *Pseudomonas* peritonitis is often characterized by severe symptoms, difficult treatment, and poor prognosis, and is closely related to catheter-related and iatrogenic factors (6). Catheter infection should be suspected in all patients with peritonitis caused by this type of pathogen, and the catheter needs to be removed for the infection to be cured (7). Despite its significance, research and reports on *pseudomonas* peritonitis in mainland China are lacking. Therefore, in this study, we aimed to describe the clinical features, antibiotic regimens, antibiotic resistance, and outcomes in a 15-year cohort of peritoneal dialysis patients in southern China and summarize our experience in the treatment of *pseudomonas* peritonitis in clinical work.

## MATERIALS AND METHODS

### Study population

Patients receiving PD at the First Affiliated Hospital of Sun Yat-sen University and Foshan First People’s Hospital from 1 January 2008 to 31 December 2022 were included in this study. The follow-up period ended on 31 January 2023. Requirements for study inclusion were (i) age ≥18 years old and PD treatment for more than 3 months; (ii) peritonitis patients whose peritoneal dialysis effluent (PDE) culture contains *Pseudomonas* or with culture-negative, positive metagenomic next-generation sequencing showing *Pseudomonas*.

### Definitions

Peritonitis was defined as meeting two of the following three criteria: (i) clinical features consistent with peritonitis, abdominal pain, and/or turbid dialysate fluid; (ii) dialysate fluid white blood cell count >100/μL or polymorphonuclear leukocyte (PMN) ratio >50%; and (iii) PDE culture or metagenomic next-generation sequencing is positive. Relapse was defined as peritonitis with the same pathogen occurring within 4 weeks of the previous peritonitis or culture-negative peritonitis. Death-censored technique failures were defined as a switch to HD at least 3 months for any reasons. Peritonitis-related death was defined as death caused by septic shock in patients with active peritonitis or death within 4 weeks after the onset of peritonitis (8). Exit site infection (ESI) was defined as meeting one of the following criteria: (i) The outlet of the catheter is continuously accompanied by redness and swelling; (ii) abnormal secretions at the catheter exit or tunnel; (iii) pain, tenderness at the exit or pain at the tunnel site; and (iv) positive bacterial culture of the secretions. The occurrence of one of the above four items is a diagnosis of catheter exit infection (9). Primary response was defined as abdominal pain relief on the fifth day of antibiotics therapy, dialysate clarification, and PDE neutrophil count <100/μL. Complete cure was defined as complete resolution of peritonitis with antibiotics alone, and no recurrence or recurrent episodes within 4 weeks of completion of treatment (10). Treatment success was defined as complete resolution of peritonitis after treatment, without removal of the Tenckhoff tube, and no recurrence within 30 days. Treatment failure was defined as treatment failure including discontinuation of PD, need for hemodialysis (HD) treatment, whether temporary or permanent, and death during peritonitis (11).

### Clinical management of peritonitis

After collecting PDE samples, patients who met the diagnostic criteria for peritonitis received initial intraperitoneal (IP) antibiotics: first-generation cephalosporins or vancomycin to cover Gram-positive bacteria and third-generation cephalosporins or aminoglycosides to cover Gram-negative bacteria. The initial IP antibiotic regimen was evaluated and modified when drug susceptibility results were available. When the culture result was *Pseudomonas* (some patients could choose to undergo genetic sequencing testing at the same time), the patient would receive at least 3 weeks of combined agent with activity against *Pseudomonas* covering Gram-negative bacteria such as ceftazidime combined with amikacin. In patients with poor response, refusal to remove the catheter, persistent abnormality infection indicators, or septic shock, a third antibiotic would be added after consultation with the nephrologist and the infectious disease department. After effective antibiotic treatment, if the white blood cell count did not decrease significantly after 5 days, the patient would be advised to remove the Tenckhoff catheter and transfer to HD.

### Microbiology testing

Of the collected PDE (which must be kept in the abdomen for at least 2 h), 5–10 mL was infused into Bact/Alert anaerobic and aerobic bottles (BioMérieux, Durham, NC, USA). All isolates' species and antibiotic susceptibility were evaluated using the VITEK-2 Auto Microbic system (BioMérieux, St. Louis, MO, USA) (12). Twenty milliliters of aseptically collected PD effluent samples (which need to be retained in the abdomen for more than 2 h) was placed into a disposable, sterile, airtight, nuclease-free, and other nucleic acid amplification inhibitor-free container, and next-generation sequencing of metagenomic genome was performed using an MGISEQ-2000 gene sequencer (13).

### Clinical data collection

We reviewed the demographic data of all PD patients, including age, gender, presence of diabetes mellitus (DM), primary renal disease, residual renal function, body mass index (BMI), number of previous peritonitis, tunnel and catheter exit infections, and age on the initiation of dialysis. Identification of *Pseudomonas* to the species level, antibiotic susceptibility, baseline laboratory data, and clinical data 1 month before the onset of peritonitis were compared between the successful treatment and treatment failure groups, including nutritional status [triglycerides, total cholesterol, high-density lipoprotein cholesterol (HDL-C), low-density lipoprotein cholesterol (LDL-C), serum albumin, hemoglobin] and residual renal function (eGFR, urine output). The status of antibiotic resistance and use were summarized after peritonitis episodes.

### Statistical analysis

The data that fit the normal distribution were expressed as the mean standard deviation, and *t*-test was used to compare groups. The data that did not fit the normal distribution were compared with M (Q1–Q3), and the nonparametric rank sum test was used to compare groups. The counting data were reported as a rate, and Chi-Square Test was performed to compare groups. *P* < 0.05 was considered statistically significant. The incidence of *Pseudomonas* was estimated as follows: number of *Pseudomonas* occurrences over time/dialysis year of infection risk was estimated as the number of occurrences per year, and repeated peritonitis was calculated as a single event.

## RESULTS

**Fig 1:**
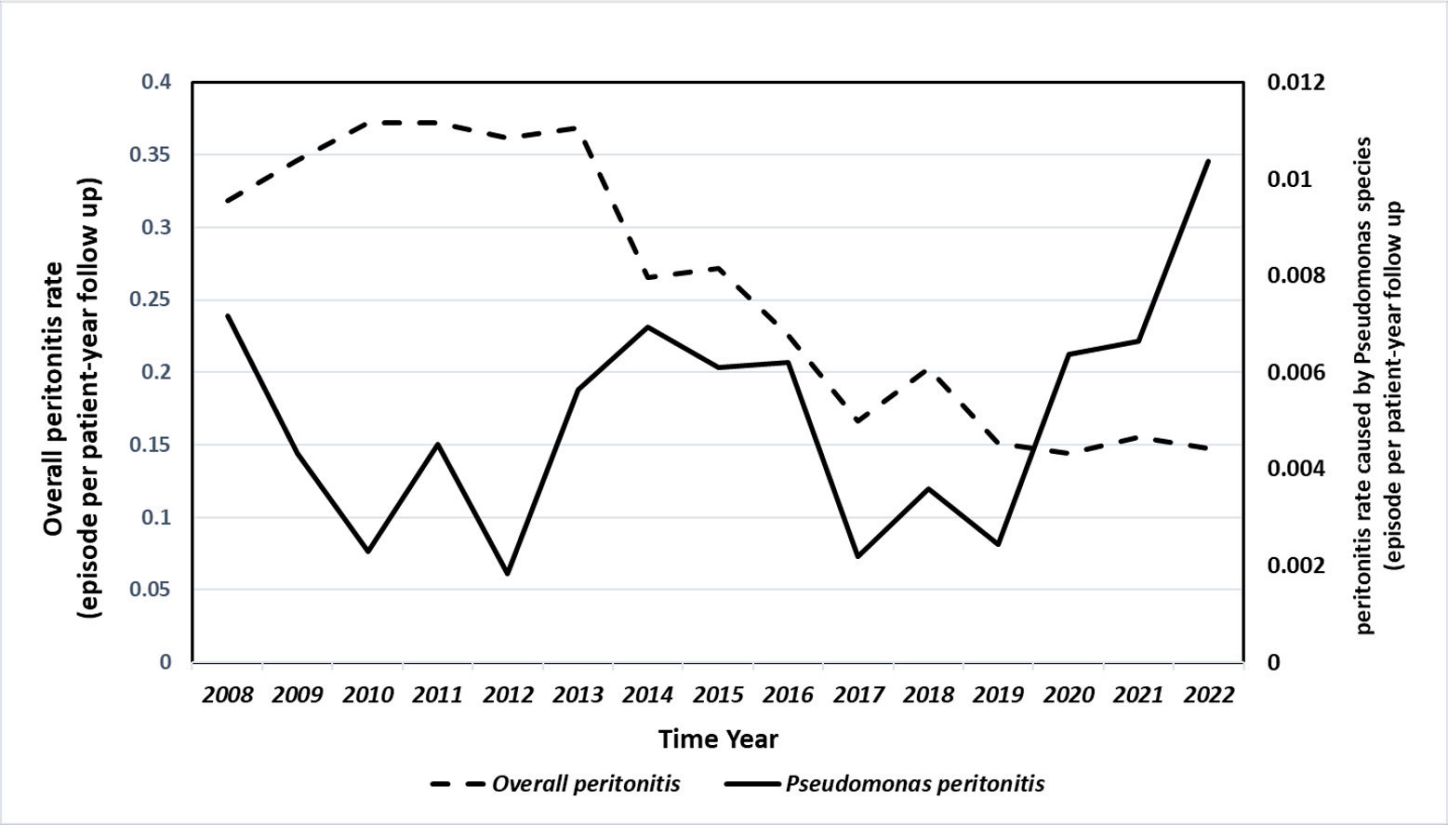
Evolution in incidence of *Pseudomonas* peritonitis and overall peritonitis from 2008 to 2022.

A total of 3,459 PD patients were included. During the follow up of 14,035.4 person-year, 2,242 episodes of peritonitis were confirmed, among which 57 peritonitis cases were caused by *pseudomonas*. Fifty-three cases showed *Pseudomonas* in ascitic fluid culture, and four cases were confirmed by gene sequencing. The incidence rate of the overall peritonitis was 0.16 episode per patient-year and *Pseudomonas* peritonitis was 0.0041 episode per patient-year. Figure 1 summarizes the changes in the incidence of all peritonitis and *Pseudomonas* peritonitis during the follow-up period. Although the overall number of peritonitis episodes declined, the incidence of *Pseudomonas* peritonitis had increased significantly since 2019.

The average age of patients with *Pseudomonas* peritonitis was 49.9 ± 13.6 years old, and the average dialysis time was 35.6 (0.26–129.4) months. There were 38 male patients, primary glomerulonephritis was the main primary disease in 75.4% (43 cases), 28.1% (16 cases) of patients were accompanied by catheter-related infections, and 24.6% (14 cases) of patients had peritonitis more than three times. All patients had turbid ascites and abdominal pain when seeking treatment, 8.0% and 26.3% of patients had symptoms such as vomiting and fever, 15.7% and 14.0% of patients had gastrointestinal symptoms such as diarrhea and intestinal obstruction, and 7% of patients had hypotension. All baseline demographic and clinical data are summarized in Table 1. The pathogenic bacteria were *Pseudomonas aeruginosa* in 48 cases (84.2%), *Pseudomonas putida* in 3 cases (5.3%), *Pseudomonas fluorescens* in 2 cases (3.5%), *Pseudomonas oryzae* in 1 case (1.8%), 1 case of *Pseudomonas flavus* (1.8%), 1 case of *Pseudomonas stutzeri* (1.8%), and 10 cases of mixed pathogenic bacteria (17.5%).

**TABLE 1:** Demographic characteristics and clinical findings of the study population (*n* = 57)^*a*^

Characteristics	Value
Age (years)	49.9 ± 13.6
Gender (male/female)	38/19
Diabetes [n, (%)]	43 (17.5%)
Etiology of ESRD [n, (%)]	
Glomerulonephritis	43 (68.0%)
Diabetic nephrology	10 (24.0%)
Obstructive nephropathy	1 (0.4%)
Purpura nephritis	1 (0.4%)
Lupus nephritis	1 (0.4%)
Polycystic kidney	1 (0.4%)
Baseline eGFR (ml/min/1.73 m2)	4.49 (1.84–9.69)
Baseline BMI (kg/m2)	22.1 ± 2.97
Peritonitis more than 3 times	14 (24.6%)
Tunnel & exit site infection	16 (28.1%)
PD vintage (months)	35.6 (0.26–129.4)
Sign or symptom	
Cloudy Dialysis Effluent	57 (100%)
Abdominal pain	57 (100%)
Fever	15 (26.3%)
Diarrhea	9 (15.7%)
Hypotension	4 (7.0%)
Intestinal obstruction	8 (14.0%)

^*a*^ Data are presented as frequencies and percentages for categorical variables, mean ± SD for normally distributed continuous variables, or median (25th; 75th percentiles) for non-normally distributed continuous variables. Abbreviations: ESRD, end-stage renal disease; eGFR, estimated glomerular filtration rate; BMI, body mass index; CCI, Charlson Comorbidity Index; PD, peritoneal dialysis.

The antibiotic susceptibility of *Pseudomonas* to commonly used Gram-negative antibiotics is shown in Table 2. Approximately 89.4% of *Pseudomonas* was sensitive to ceftazidime, followed by 87.7% of amikacin, and the resistance rate to levofloxacin is 10.5%. *Pseudomonas* was susceptible to ceftazidime, cefepime, imipenem, meropenem, and piperacillin/tazobactam. The course of antibiotic treatment is shown in Table 3. The most commonly used first-line antibiotics covering Gram-negative bacteria include ceftazidime (27 cases, 47.3%), amikacin (28 cases, 49.1%), and netilmicin (2 cases, 3.5%); 50.8% (29 cases) of the patients used a combination of two antibiotics covering Gram-negative bacteria, and the most commonly used antibiotic combination covering Gram-negative bacteria was ceftazidime combined with amikacin (18 cases, 31.6%). In 13 cases (22.8%), the third antibiotic was used according to different clinical status. The most common third antibiotics were carbapenems (7 cases) and oral ciprofloxacin (4 cases). A total of 13 patients were treated with 3 kinds of Gram-negative antibiotics, of which 4 cases were treated successfully.

**TABLE 2:** Antibiotic susceptibility of *Pseudomonas* species isolate from effluent (*n* = 57)

Antibiotics	Susceptible (%)	Intermediate (%)	Resistant (%)	Not tested (%)
Ciprofloxacin	84.2	1.7	3.5	10.5
Amikacin	87.7	0.0	1.7	10.5
Imipenem	84.2	5.2	0.0	10.5
Cefepime	80.7	5.2	0;0	14.0
Ceftazidime	89.4	0.0	0.0	10.5
Piperacillin/tazobactam	85.9	3.5	0.0	10.5
Levofloxacin	71.9	7.0	10.5	10.5
Meropenem	80.7	0.0	0.0	19.2
Cefoperazone/sulbactam	70.1	10.5	1.7	17.5

**TABLE 3:** Summary of antibiotic therapy (*n* = 57)^*a*^

First empirical Gram-negative coverage	No. of cases (%)	Second anti-*Pseudomonas* antibiotics	No. of cases (%)
Ceftazidime	27 (47.4%)	Amikacin	13 (22.8%)
		Cefoperazone/sulbactam	2 (3.5%)
		Levofloxacin	2 (3.5%)
		Moxifloxacin	3 (5.2%)
		Ciprofloxacin	1 (1.8%)
		Meropenem	1 (1.8%)
		-	5 (8.8%)
Amikacin	28 (49.1%)	Ciprofloxacin	2 (7.0%)
		Ceftazidime	5 (8.8%)
		Imipenem	2 (3.5%)
		Cefoperazone/Sulbactam	7 (12.2%)
		Levofloxacin	1 (1.8%)
		Cefepime	1 (1.8%)
		-	10 (17.5%)
Etimicine	2 (3.5%)	Amikacin	1 (1.8%)
		Cefoperazone/sulbactam	1 (1.8%)

^*a*^ -, no use of the second anti- *Pseudomonas* antibiotic.

**Fig 2:**
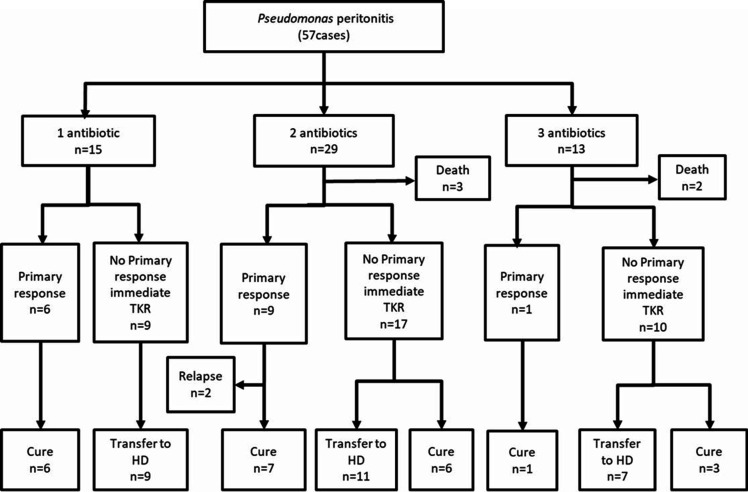
Summary of clinical outcome. (Abbreviations: TKR, Tenckhoff catheter removal; PD, peritoneal dialysis HD, hemodialysis).

The outcomes of clinical treatment are summarized in Fig. 2. The overall primary response rate was 28.1% (16 cases), and complete cure rate was 40.4% (23 cases). The complete cure rate (55.6% vs 36.7%; *P* = 0.15) between episodes receiving ceftazidime and other antimicrobials as initial antibiotic regimen or using the three antimicrobial regimen and other antibiotic regimen (38.4% vs 43.2%; *P* = 0.22) had no significant difference. Compared to failed response cases, the patients in the successful treatment group had higher albumin levels (35.9 ± 6.2; *P* = 0.008) and residual urine volume (650.7 ± 375.5; *P* = 0.04) (Table 4).

**TABLE 4:** The differences in characteristics between the treatment failure group and the treatment success group during the observation period^*a*^

Variables	Treatment failure group (*n* = 34)	Treatment success group (*n* = 23)	*P* value
Age (years)	50.4 ± 12.1	49.4 ± 15.3	0.54
Men (%)	22(65)	17(74)	0.26
Vintage (months)	37.4 ± 35.8	33.6 ± 30.5	0.93
Nutritional status			
TC (μmol/L)	4.1 ± 1.2	4.1 ± 1.00	0.54
TG (μmol/L)	1.4 ± 0.8	1.29 ± 0.98	0.78
HDL-c (μmol/L)	1.2 ± 0.4	1.44 ± 1.66	0.57
LDL-c (μmol/L)	2.3 ± 1.0	2.36 ± 0.81	0.64
ALB (g/L)	34.6 ± 4.4	36.9 ± 6.2	0.008
HGB (g/L)	100.6 ± 19.6	99.1 ± 19.0	0.96
Dialysis adequacy			
Total Kt/V	1.93 (1.5–2.13）	1.94 (1.65–2.16）	0.53
Residual eGFR (mL/min/1.73 m^2^)	4.50 (3.43–5.01）	4.48 (2.92–5.79）	0.84
Urine output (L/day)	581.7 ± 580.8	650.7 ± 584.8	0.04

^*a*^ Data are presented as frequencies and percentages for categorical variables, mean ± SD for normally distributed continuous variables, or median (25th; 75th percentiles) for non-normally distributed continuous variables. Abbreviations: ALB, albumin; HGB, hemoglobin; TC, total cholesterol; TG, triglyceride; LDL-C, low-density lipoprotein cholesterol; HDL-C, high-density lipoprotein cholesterol.

A total of 34 (57.9%) patients had failed response after treatment, among which, 2 cases (3.5%) were relapses, 27 cases (47.4%) were converted to HD, and 5 cases (8.8%) died. The causes of death were septic shock (1 case), sudden cardiac arrest (1 case), cerebral hemorrhage (1 case), malignant tumor (1 case), and gastrointestinal bleeding (1 case).

## DISCUSSION

This study revealed that the incidence rate of peritonitis related to *Pseudomonas* was 0.0041 episodes per person-year. Following treatment, only 40.2% of patients were able to continue receiving PD. Patients in the successful treatment group have higher albumin levels and residual urine output. The drug susceptibility indicated that *Pseudomonas* was still highly sensitive to the commonly used antibiotics ceftazidime and amikacin, which cover Gram-negative bacteria. There was no significant difference in the complete cure rate of peritonitis between the combination of three antibiotics and other antibiotics regimen.

In our study, the incidence of *Pseudomonas* peritonitis was very low, which was lower than previous results from Hong Kong (0.04 episode per patient-year) and Australia (0.032 episode per patient-year) (6, 14). However, this gap was significantly narrowed when comparing the incidence of peritonitis, which may be related to the fact that the positive rate of PDE culture in our center was only 73.8% (15). Although the overall peritonitis continues to decline, the incidence of *Pseudomonas* peritonitis had increased significantly since 2019, which might be related to the application of new diagnostic technology and the increase in culture positivity rate.

Recent studies in Hong Kong have shown that the incidence rate of *Pseudomonas* peritonitis in Hong Kong was 0.04 episodes per person-year, *Pseudomonas aeruginosa* was dominant (accounted for 92.1% of *Pseudomonas* species), and they believed that the warm and humid climate was conducive to the colonization of *Pseudomonas* (14). In our study, the proportion of *Pseudomonas aeruginosa* exceeded 80%, which was similar to reports in Hong Kong because Guangzhou and Hong Kong have similar climates. Although many previous studies indicated that season has no relationship with the incidence of peritonitis (16, 17), some studies have shown that higher temperature and humidity were associated with a significant increase in the incidence of Gram-negative bacteria in summer, and the prognosis of peritonitis occurring in summer was poor (18).

Relevant studies have shown that the proportion of fever, abdominal pain, and peritoneal dialysate turbidity in the Gram-negative bacteria group was significantly higher than that in the Gram-positive bacteria group (19). The initial clinical manifestations of *Pseudomonas* peritonitis were very severe, with more pronounced diarrhea and abdominal pain, which were thought to be associated with higher manifestations of ascites, reflecting the greater virulence of *Pseudomonas* (20). All patients in our study had symptoms of abdominal pain, turbid ascites, and even hypotension.

*Pseudomonas aeruginosa* is easy to colonize in a PD catheter, and it is easy to form a biofilm, which enhances drug resistance. Recent studies have shown that extracellular DNA (eDNA) and extracellular polysaccharides (Psl), the two main components of *Pseudomonas aeruginosa* biofilm matrix, form Psl-eDNA fiber network on the biofilm, which increases the colonization ability and drug resistance of *Pseudomonas aeruginosa* (21). The close relationship between *Pseudomonas* and catheter-related infections has been reported many times (22); 28% of *Pseudomonas* peritonitis patients in our study were complicated with catheter-related infection. Approximately 13.1% of patients in Hong Kong were complicated with outlet infection (14). Although the international society for peritoneal dialysis (ISPD) guidelines state that the symptoms of catheter-related infections are not completely relieved with, e.g., a 3-week course of treatment, changing the tunnel and outlet location and replacing the tube at the same time of anti-infective treatment, given the poor prognosis of *Pseudomonas* peritonitis, may lead to earlier extubation, and removal of the source of infection can better help patients avoid abdominal infection and better protect peritoneal function.

The main resistance mechanisms of *Pseudomonas* to antibiotics were mainly related to the following three aspects: inherent resistance, acquired resistance, and adaptive resistance (23, 24). The spectrum of drug-resistant bacteria in different centers may be different. In recent studies in Hong Kong, due to the widespread use of antibiotics, the resistance of *Pseudomonas aeruginosa* to commonly used ceftazidime and gentamicin increased year by year compared with that of 1995–1999 (14). Related studies in Germany have shown that the resistance of Gram-negative bacteria to the third-generation cephalosporins has gradually increased (25). According to a 10-year experience at Suzhou University, the sensitivity of Gram-negative bacteria to ceftazidime has decreased from 100% to 84%. There was no significant change in the sensitivity and drug resistance of gentamicin (19). However, according to reports in Taiwan, only 8% of the 892 *Pseudomonas aeruginosa* isolates reduced their sensitivity to ceftazidime (26). However, in our drug sensitivity statistics, ceftazidime, ciprofloxacin, and aminoglycosides still have high susceptibility rates to *Pseudomonas*. We believe that it may be due to the physical mechanism of biofilm that limits the penetration of antibiotics, so it will show that the drug sensitivity adaptation in ascites was sensitive, but the therapeutic effect was not the idea.

The 2022 ISPD guidelines recommend two different mechanisms of Gram-negative antibiotics (ceftazidime/cefepime + gentamicin/tobramycin/amikacin or ciprofloxacin) for *pseudomonas* and pull out the tube after 5 days of ineffective use of antibiotics (8), but in the course of treatment, due to the types of drugs and the course of treatment restrictions, many patients did not have time to use two Gram-negative antibiotics. According to the Australian study, only approximately 21% of *Pseudomonas* peritonitis patients received combined treatment, and the most commonly used combination was ciprofloxacin and gentamicin (6). Eighty-five percent of patients in Hong Kong were treated with a combination of ceftazidime and gentamicin (14). Ceftazidime and amikacin were the most commonly used combination of antibacterial regimens in this study. Carbapenem antibiotics or ciprofloxacin were selected as the third antibiotics. However, our research and that of Hong Kong all show that the effect of using a combination of three antibiotics does not seem to have an advantage (although there may be a bias caused by the disease), and there was evidence that carbapenem was as effective in the treatment of PDAP as ceftazidime combined with netilmicin (27). In addition, ceftazidime–avibactam, a new antibiotic for *Pseudomonas*, has been approved for marketing in China, and high susceptibility rate to *Pseudomonas aeruginosa* provides more options for patients with limited antibiotic options (28).

In our study, the overall primary response rate was 28.1%, and complete cure rate was 40.4%. We found that better albumin levels and residual urine volume were associated with better treatment outcomes. Many previous studies have also confirmed that the prognosis of peritonitis was related to the patient’s nutritional level and residual renal function (29). In a related study from Hong Kong, residual glomerular filtration rate (GFR) and normal protein nitrogen appearance significantly decreased after an episode of *Pseudomonas* peritonitis, but there were no significant changes in total Kt/V or other nutritional parameters (14). We believe that patients with better residual renal function, who tend to have better nutritional status due to better appetite and toxin removal, may be better able to cope with the onslaught of peritonitis.

Our research has several limitations. First, this study is a dual-center retrospective study with a relatively small sample size. Second, our current experience in the treatment of *Pseudomonas* peritonitis is preliminary. In addition, drug sensitivity is related to the center’s long-term experience in the use of antibiotics, and the results may not be applicable to other centers.

### Conclusion

Although the incidence of PD-related peritonitis caused by *Pseudomonas* in South China was low, the symptoms were serious, and the therapeutic effect was not ideal despite the high sensitivity of antibiotics. The patients with successful treatment have higher albumin level, better nutritional status, and high residual rate of renal function, which may lead to a better prognosis. Further research is needed to increase the understanding of the virulence and drug resistance mechanism of *Pseudomonas* peritonitis, to improve the therapeutic effect of *pseudomonas* peritonitis and the technique survival of PD patients.

## Data Availability

The original data supporting the conclusions of this article will be provided by the author without reservation.
